# A functional genomics screen for microRNA regulators of NF-kappaB signaling

**DOI:** 10.1186/1741-7007-11-19

**Published:** 2013-02-28

**Authors:** Anthony O Olarerin-George, Lauren Anton, Yih-Chii Hwang, Michal A Elovitz, John B Hogenesch

**Affiliations:** 1 Genomics and Computational Biology Graduate Group, 1420 Blockley Hall, 423 Guardian Drive, Perelman School of Medicine at the University of Pennsylvania, Philadelphia, PA 19104, USA; 2 Maternal and Child Health Research Program, Department of Obstetrics and Gynecology, 1354 Biomedical Research Building II/III, 421 Curie Blvd., Perelman School of Medicine at the University of Pennsylvania, Philadelphia, PA 19104, USA; 3 Department of Pharmacology and the Institute for Translational Medicine and Therapeutics, Smilow Translational Research Center 10-124, 3400 Civic Center Blvd., Bldg. 421, Perelman School of Medicine at the University of Pennsylvania, Philadelphia, PA 19104, USA

**Keywords:** microRNA, miRNA, miR-517, NF-kappaB, TNIP1, TNF, RNAi, screen, apoptosis

## Abstract

**Background:**

The nuclear factor-KappaB (NF-κB) pathway is conserved from fruit flies to humans and is a key mediator of inflammatory signaling. Aberrant regulation of NF-κB is associated with several disorders including autoimmune disease, chronic inflammation, and cancer, making the NF-κB pathway an attractive therapeutic target. Many regulatory components of the NF-κB pathway have been identified, including microRNAs (miRNAs). miRNAs are small non-coding RNAs and are common components of signal transduction pathways. Here we present a cell-based functional genomics screen to systematically identify miRNAs that regulate NF-κB signaling.

**Results:**

We screened a library of miRNA mimics using a NF-κB reporter cell line in the presence and absence of tumor necrosis factor (+/- TNF). There were 9 and 15 hits in the -TNF and +TNF screens, respectively. We identified putative functional targets of these hits by integrating computational predictions with NF-κB modulators identified in a previous genome-wide cDNA screen. miR-517a and miR-517c were the top hits, activating the reporter 86- and 126-fold, respectively. Consistent with these results, miR-517a/c induced the expression of endogenous NF-κB targets and promoted the nuclear localization of p65 and the degradation of IκB. We identified TNFAIP3 interacting protein1 (TNIP1) as a target and characterized a functional SNP in the miR-517a/c binding site. Lastly, miR-517a/c induced apoptosis *in vitro*, which was phenocopied by knockdown of TNIP1.

**Conclusions:**

Our study suggests that miRNAs are common components of NF-κB signaling and miR-517a/c may play an important role in linking NF-κB signaling with cell survival through TNIP1.

## Background

Nuclear factor kappa-B (NF-κB) is well conserved, ubiquitously expressed, and a pivotal regulator of immune response, inflammation and cell survival [1-3]. Dysregulation of NF-κB signaling is associated with numerous human diseases ranging from cancer to Crohn's disease [4], underscoring the role of NF-κB in physiological processes. Since the discovery of NF-κB twenty-six years ago [5,6], much insight has been gained into the regulation of its signaling and many pathway components have been characterized [7].

The NF-κB transcription factor is a dimer composed of structurally related Rel family proteins [7]. Of these, the p50-p65 NF-κB heterodimer is best characterized. The cytokine tumor necrosis factor (TNF) is a strong inducer of p50-p65/NF-κB signaling [7,8]. Upon binding to cell surface receptors, TNF initiates a signaling cascade resulting in the phosphorylation of I-kappaB (IκB) kinase (IKK) -a large cytoplasmic multi-protein complex [7,9]. IKK in turn phosphorylates IκB-α bound to NF-κB. This results in proteasomal degradation of IκB-α, freeing NF-κB to translocate into the nucleus and activate its targets [7]. Many of these targets include activators (for example, TNF, IL6) and inhibitors (for example, IκB-α, A20, tumor necrosis factor alpha-induced protein 3 interacting protein 1 (TNIP1)) of NF-κB signaling, hence providing context specific feedback regulation.

MicroRNAs (miRNAs) are a class of small non-coding RNAs (ncRNAs) that bind to target mRNAs and promote transcript degradation and/or inhibit translation [10]. Studies suggest that miRNAs are common components of signal transduction pathways, conferring robustness to gene expression and feedback to cellular networks [11-13]. Several miRNAs are known to regulate NF-κB signaling [14]. The earliest example is miR-146, an inhibitor of toll-like receptor (TLR) mediated NF-κB signaling [15]. Another example, the miR-125 family, promotes NF-κB signaling by targeting TNF alpha-induced protein 3 (TNFAIP3), an inhibitor of NF-κB signaling [16]. Further exemplifying the complexity of regulation, miR-21 promotes NF-κB signaling via the PTEN/AKT pathway in MCF-10A cells [17] but inhibits lipopolysacharride (LPS) mediated NF-κB signaling in TLR4 expressing HEK293 cells [18]. In the above examples, the miRNAs are themselves direct (miR-146, miR-125ab) or indirect (miR-21) targets of NF-κB, suggesting that feedback regulation is common [15,17,19].

In this study, we sought to systematically characterize the role of miRNAs in NF-κB signaling using high-throughput cell based screening. We confirmed previously known regulators and identified novel candidates. Further, we derived likely targets for these hits by incorporating other high-throughput/computational datasets. Lastly, we characterized the top hits, miR-517a/c, identified their target, and explored the relationship between their impact on NF-κB signaling and their regulation of apoptosis in the human embryonic kidney cell line HEK293 and the osteosarcoma cell line U-2 OS.

## Results

### Genome-wide miRNA screen identifies regulators of NF-κB signaling

**Figure 1:**
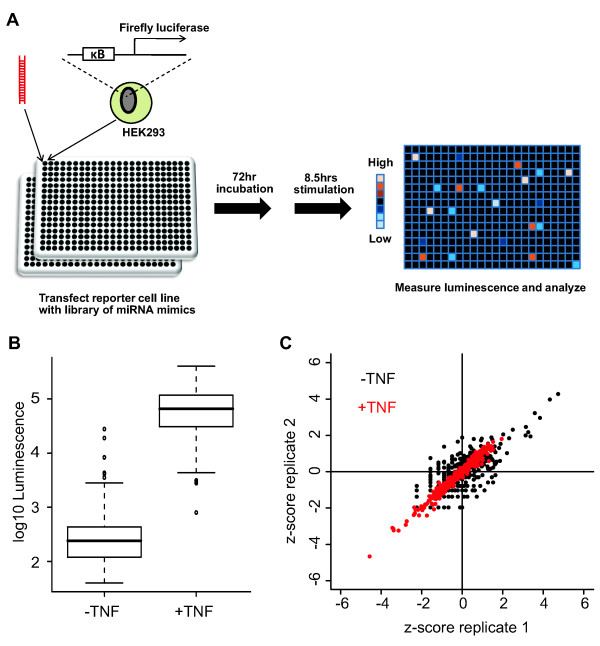
**Genome-wide miRNA screen for regulators of NF-κB signaling**. **(A) **Primary screen schematic. HEK293 cells stably expressing a NF-κB-luc reporter were transfected with a library of miRNA mimics. Plates were incubated under normal growth conditions for 72 hours. Cells were then treated with vehicle control or TNF for 8.5 hours. Luminescence was read from each well. **(B) **Boxplots of luminescence values for the -TNF and +TNF screens. **(C) **Replicate analysis for each screen (-/+TNF) with luminescence values converted to Z-scores. NF-κB, nuclear factor kappa-B; NF-κB-luc, NF-κB responsive luciferase reporter; TNF, tumor necrosis factor.

To identify miRNAs involved in the regulation of NF-κB signaling, we transfected HEK293 cells stably expressing a NF-κB responsive luciferase reporter (NF-κB-luc cells) with a library of 328 miRNA mimics (miRBase v8.0; see Additional file 1, Table S1 for annotation) in duplicate (Figure 1A). Seventy-two hours post-transfection, the cells were treated with TNF (+TNF) or vehicle control (-TNF) for 8.5 hours. Luminescence was then measured from each well (Figure 1A; Additional file 1, Table S2). Boxplots of the luminescence values for each screen are shown in Figure 1B. The addition of TNF to the cells induced the average plate-wide reporter activity approximately 400-fold (Figure 1B). *Z*-scores were calculated from the luminescence values (see methods) and were highly replicable for the -TNF (r = 0.73) and +TNF (r = 0.98) screens (Figure 1C). Hits from the screens were those miRNAs that differentially regulated the reporter (up or down) relative to the plate median. At a *P*-value cutoff of 0.02 there were 9 (false discovery rate (FDR) = 18%) and 15 (FDR = 12%) hits in the -TNF and + TNF screens, respectively (Wilcoxon rank-sum test; Table 1). Two of these, miR-483 and miR-452*, were hits in both screens.

**Table 1:** Top hits in -TNF and +TNF screens.

-TNF screen				
**miRNA**	**Seed sequence**	**Fold change**	***Z*-score**	***P*-value**

hsa-miR-517c	UCGUGCA	126.3	4.8	0.015
hsa-miR-517a	UCGUGCA	86.2	4.4	0.015
hsa-miR-544	UUCUGCA	37.9	3.6	0.016
hsa-miR-337	CCAGCUC	38.0	3.6	0.016
hsa-miR-452*	CAGUCUC	19.7	3.0	0.018
hsa-miR-326	CUCUGGG	18.8	2.9	0.018
hsa-miR-184	GGACGGA	19.0	2.8	0.019
hsa-miR-34b	AGGCAGU	16.1	2.7	0.019
hsa-miR-483	CACUCCU	12.8	2.5	0.019

**+TNF screen**				

**miRNA**	**Seed sequence**	**Fold change**	***Z*-score**	***P*-value**

hsa-miR-124a	UAAGGCA	-82.8	-4.6	0.015
hsa-miR-452*	CAGUCUC	4.5	1.6	0.015
hsa-miR-483	CACUCCU	6.1	1.9	0.015
hsa-miR-506	AAGGCAC	-22.8	-3.3	0.016
hsa-miR-345	GCUGACU	-22.9	-3.3	0.016
hsa-miR-24	GGCUCAG	-23.7	-3.3	0.016
hsa-miR-375	UUGUUCG	-21.2	-3.2	0.016
hsa-miR-141	AACACUG	3.9	1.4	0.018
hsa-miR-130a	AGUGCAA	3.8	1.4	0.018
hsa-miR-491	GUGGGGA	-15.3	-2.9	0.018
hsa-miR-30a-3p	UUUCAGU	3.9	1.4	0.018
hsa-miR-489	GUGACAU	-13.7	-2.7	0.018
hsa-miR-125b	CCCUGAG	3.5	1.3	0.020
hsa-miR-200b	AAUACUG	3.8	1.4	0.020
hsa-miR-210	UGUGCGU	-9.8	-2.4	0.020

miRNA, microRNA; TNF, tumor necrosis factor.

### Putative functional targets of miRNAs derived from overlap of prediction data and genome-wide cDNA screen

**Figure 2:**
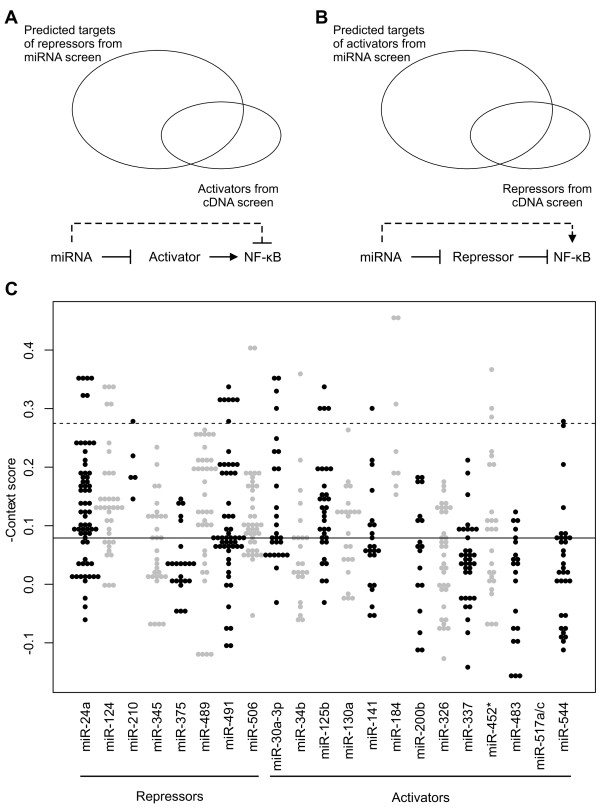
**Functional target identification for top miRNA hits**. (A and B) Predicted miRNA targets were compared to NF-κB signaling modulators identified in a previous genome-wide cDNA screen. **(A) **miRNAs that repressed NF-κB likely targeted activators of the pathway. **(B) **Conversely, miRNAs that activated NF-κB likely targeted repressors. **(C) **Scatterplot of the context scores of the predicted targets for each miRNA hit from the screen. y-axis is the negated context score (a more negative context score indicates a more confident prediction). Colors are alternated between black and grey for visual clarity. Solid black line is the median -context score for all miRNA-mRNA predictions (that is all 328 miRNAs and approximately 31,000 transcripts). Dashed line represents cutoff of 2 MAD from the median. MAD, median absolute deviations; miRNA microRNA; NF-κB, nuclear factor kappa-B.

In a previous genome-wide cDNA screen for regulators of NF-κB signaling, we identified 154 activators and 88 repressors [20]. To identify functional targets of the miRNA hits in our screen, we compared regulators from the cDNA screen with predicted miRNA targets. The predictions were generated with code from TargetScan 6.0 [21,22]. For miRNAs that activated signaling in our screen, we took the intersection of predicted targets and negative regulators from the cDNA screen (Figure 2A). Conversely, for miRNAs that repressed signaling, we took the overlap between predicted targets and activators from the cDNA screen (Figure 2B). We obtained several candidate genes for each of the miRNAs with the exception of miR-517a/c (Figure 2C). This was due in part to the smaller number of total predicted targets for miR-517a/c compared to the other miRNAs (see Additional file 1, Table S3). To obtain high confidence functional targets for each of the miRNAs, we set a cutoff of two median absolute deviations (mad) from the median prediction score of all miRNA-mRNA interactions (Figure 2C). The prediction score, or context score (CS), is the sum of multiple feature scores characterizing the proposed miRNA-mRNA interaction [21]. The more negative the CS, the more favorable the predicted interaction. The resulting miRNAs and their targets are listed in Table 2. Five of the 8 miRNA repressors and 7 of the 13 miRNA activators had one or more predicted targets meeting the cutoff. miR-124 and miR-506 were particularly noteworthy. They both repressed NF-κB signaling in our screen and were predicted to target RELA/p65, a core pathway component and a positive regulator in the cDNA screen (Table 2). Also, miR-452*, an activator in our screen, was predicted to target NFKBIE, a repressor of NF-κB signaling. Hence, these results suggest that the integration of data from genome-wide cDNA screens and computational target predictions may help in identifying functional miRNA targets.

**Table 2:** High confidence functional targets from miRNA predictions and genome-wide cDNA screen.

miRNAs repressing NF-κB signaling				
**miRNA**	**Gene**	**Transcripts**	**CS**	**CSP**

miR-24	MYD88	NM_001172569, NM_002468, NM_001172568, NM_001172567, NM_001172566	-0.319	95
	RASL11B	NM_023940	-0.35	96
	RELA	NM_021975, NM_001145138	-0.307	93
miR-124	TRIM22	NM_001199573, NM_006074	-0.339	96
	BECN1	NM_003766	-0.339	96
miR-210	CCNL2	NM_001039577	-0.275	85
miR-491	PLEKHB1	NM_021200, NM_001130033, NM_001130035, NM_001130034, NM_001130036	-0.314	98
	TULP3	NM_003324	-0.278	97
	TNFSF10	NM_001190943	-0.336	99
miR-506	RELA	NM_021975, NM_001145138	-0.401	98

**miRNAs activating NF-κB signaling**				

**miRNA**	**Gene**	**Transcripts**	**CS**	**CSP**

	MATN2	NM_030583, NM_002380	-0.351	96
miR-30a	GPR161	NM_153832	-0.298	93
	PTGER2	NM_000956	-0.328	95
miR-34b	TLR7	NM_016562	-0.356	98
miR-125b	IKBKG	NM_001099857, NM_001099856, NM_001145255	-0.297	92
	MAP3K11	NM_002419	-0.337	95
miR-141	PTGDR	NM_000953	-0.3	97
miR-184	TNK2	NM_001010938, NM_005781	-0.452	94
	SLC7A5	NM_003486	-0.306	85
miR-452*	LPAR2	NM_004720	-0.304	95
	STMN1	NM_001145454	-0.367	98
	NFKBIE	NM_004556	-0.288	94
miR-544	TSPAN13	NM_014399	-0.279	92

CS, context score; CSP, context score percentile; miRNAs, microRNAs; NF-κB, nuclear factor-kappa B. A more negative CS is associated with a more favorable miRNA-mRNA interaction.

### Secondary screen confirms top hits

**Figure 3:**
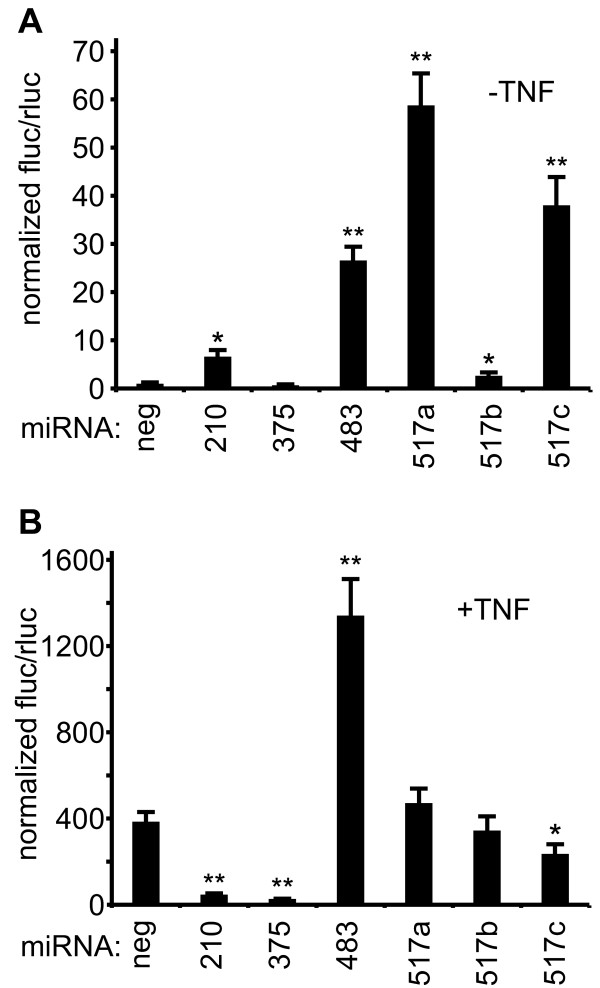
**Secondary screen validation of top hits**. NF-κB -luc cells were co-transfected with the indicated miRNAs and a constitutively expressed *Renilla *reporter plasmid. Firefly luciferase activity was normalized to that of *Renilla *luciferase. All values are means ± SD. n = 3. *P ≤0.05, **P ≤0.01. miRNAs, micro RNAs; NF-κB, nuclear factor kappa-B; NF-κB-luc, NF-κB responsive luciferase reporter; SD, standard deviation.

Next, we selected representative miRNAs for secondary screening: those that activated the reporter in the -TNF screen (miR-517a, miR-517c), inhibited the reporter in the +TNF screen (miR-210, miR-375), or activated the reporter in both screens (miR-483). The miR-517 family consists of three highly homologous members, miR-517a, miR-517b, and miR-517c, yet intriguingly, miR-517b, was not a hit in the primary screen. Nonetheless, we included miR-517b in the secondary screen to eliminate the possibility that it was a false-negative. In the secondary screen, the miRNAs were co-transfected with a constitutively expressed *Renilla *reporter plasmid. This served as a control for transfection efficiency and background effects caused by the miRNAs. Corroborating the primary screen results, miR-517a and miR-517c were potent activators (59- and 38-fold, respectively) of the reporter in the absence of TNF, but did not potentiate TNF induced reporter activity (Figure 3, A and B). In fact, there was a small but significant (*P *= 0.01) reduction in reporter activity in the miR-517c transfected cells stimulated with TNF. Overexpression of miR-517b caused a relatively small increase in reporter activity (2.6-fold) and hence, was deemed a true-negative. On the other hand, miR-210 and miR-375 overexpression significantly inhibited TNF induced reporter activity by 8.1- and 14.3-fold (*P *≤0.005, Figure 3B), respectively. Surprisingly, miR-210 induced basal reporter activity by 6.4-fold (Figure 3A; *P *= 0.02). Lastly, miR-483 potentiated signaling in both the -TNF (36-fold, *P *= 0.004) and +TNF (3.5-fold, *P *= 0.007) conditions relative to the respective negative controls (Figure 3, A and B). In all, the hits from our initial screen were validated in the secondary screen.

### Effect of miR-517 family on endogenous NF-κB targets and signaling components

We decided to follow up on the miR-517 family with additional experiments for several reasons: 1) miRNAs typically have a moderate impact on their targets yet miR-517a/c were potent activators of reporter activity in the absence of any added stimulus; 2) we were intrigued that miR-517b did not activate signaling despite extensive sequence similarity to miR-517a/c; and 3) a previous proteomics study by Luo *et al. *suggested miR-517a involvement in MAPK and TNF signaling in BeWo cells [23].

**Figure 4:**
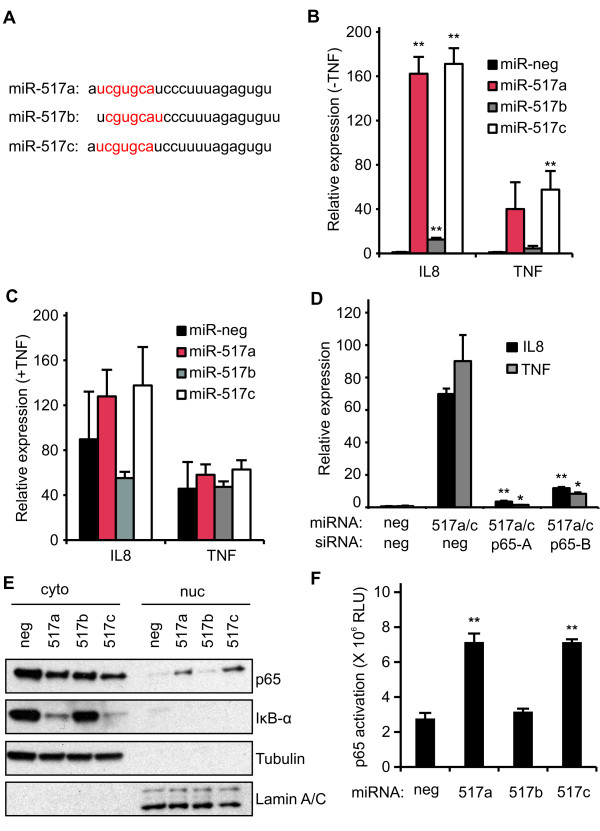
**Validation and characterization of miR-517a/c**. **(A) **Sequence comparison of the miR-517 family miRNAs. Seed sequences (nucleotides 2 to 8) are highlighted in red. (B and C) HEK293 cells were transfected with the indicated miRNA mimics for 72 hours. mRNA expression of endogenous NF-κB targets IL8 and TNF were determined with quantitative PCR (qPCR) in the absence **(B) **or presence **(C) **of TNF. Statistical comparisons are relative to the negative control. **(D) **HEK293 cells were co-transfected with the indicated miRNA mimics and siRNAs. si-p65-A and si-p65-B are two independent p65 siRNAs. Statistical comparison is relative to cells transfected with miR-517a/c + si-neg. **(E) **Western blot analysis of nuclear and cytoplasmic protein extracts from HEK293 cells transfected with the indicated miRNAs. Tubulin and Lamin A/C were cytoplasmic and nuclear loading controls, respectively. **(F) **DNA binding ELISA assay to quantify p65 activation in nuclear extracts from HEK293 cells transfected with the indicated miRNAs. All values are means ± SD. n = 3. *P ≤0.05, **P ≤0.01. miRNAs, micro RNAs; NF-κB, nuclear factor kappa-B; siRNAs, small interfering RNAs; SD, standard deviation; TNF, tumor necrosis factor.

The miR-517 family is located in a large, primate-specific miRNA cluster on human chromosome 19 (C19MC) [24]. The miR-517 miRNAs share extensive sequence homology. miR-517a and miR-517c differ by one nucleotide at position 12, and miR-517b is shifted by one nucleotide relative to miR-517a (Figure 4A). This shift in miR-517b also results in a shifted seed sequence (Figure 4A). The seed sequence of the miRNA (nucleotides 2 to 8) is a primary determinant of functional interaction between a miRNA and its target. Hence, the shift in the miR-517b seed likely alters target interaction relative to miR-517a/c. Nonetheless, it was unexpected to see such a large difference between miR-517a/c and miR-517b in our reporter assays.

Next, we wanted to test the effect of the miR-517 miRNAs on endogenous NF-κB targets. We transfected HEK293 cells with the miR-517 mimics and measured the expression of IL8 and TNF mRNA in unstimulated cells (Figure 4B) and in cells stimulated with TNF for three hours (Figure 4C). In unstimulated cells, miR-517a/c strongly induced IL8 (>160-fold) and TNF (>40-fold) expression while miR-517b had only a modest effect (13- and 4-fold, respectively), confirming the reporter results (Figure 3A). However, in cells treated with TNF, there was no significant change in IL8 and TNF expression in cells transfected with the miR-517 mimics relative to the negative control (Figure 4C). To verify that miR-517a/c were acting through the NF-κB pathway, we co-transfected the mimics with one of two independent small interfering RNAs (siRNAs) against the p65 subunit of NF-κB (efficacy of the siRNAs is demonstrated in Additional file 2, Figure S1). Both siRNAs reduced miR-517a/c induced expression of IL8 and TNF at least 6-fold (Figure 4D; *P *≤0.01). Lastly, the nuclear translocation of p65 and the proteasomal degradation of IκB-α are indicative of NF-κB activation. Hence, we evaluated the effect of miR-517a/c on the subcellular localization and expression of p65 and IκB-α in HEK293 cells. miR-517a/c, but not miR-517b, increased nuclear p65 and decreased cytoplasmic IκB-α expression relative to the negative control (Figure 4D). Further, there was increased NF-κB DNA binding activity in the nuclear extracts of cells transfected with miR-517a and miR-517c, but not miR-517b as measured with ELISA (Active Motif TransAM p65 NF-κB assay; Figure 4F). In all, these results confirmed the primary and secondary screen data that miR-517a/c, and not miR-517b, were potent activators of NF-κB signaling.

To confirm that the effects of miR-517a/c on NF-κB signaling were not restricted to HEK293 cells, we also tested primary human umbilical vein endothelial cells (HUVECs). Similarly, miR-517a/c increased target gene expression (IL8, IL6 and TNF) in a p65-dependent manner and increased p65 nuclear translocation (see Additional file 2, Figure S2).

### TNIP1 is a direct functional target of miR-517a/c

**Figure 5:**
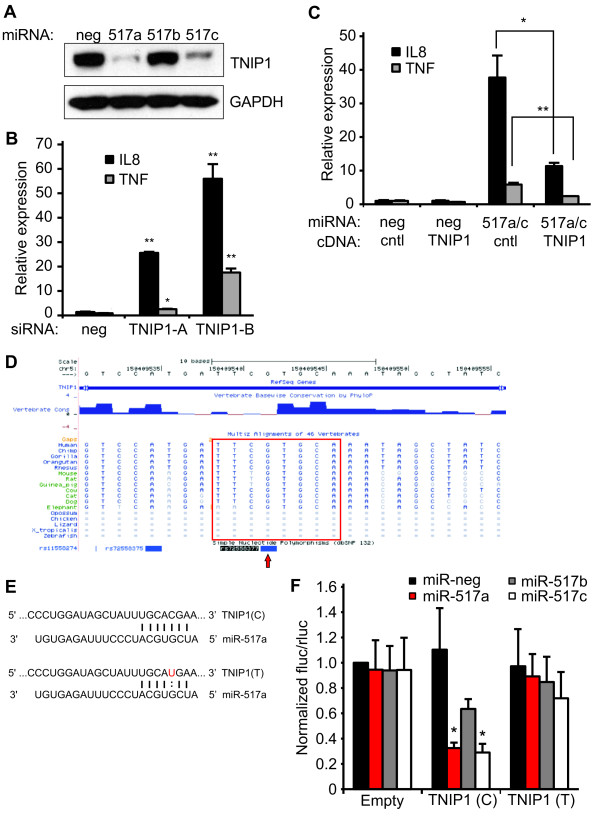
**TNIP1 is the direct functional target of miR-517a/c**. **(A) **Western blot analysis of lysates from HEK293 cells transfected with the indicated miRNAs. GAPDH was the loading control. (B and C) qPCR analysis of IL8 and TNF expression in HEK293 cells **(B) **transfected with two independent siRNAs against TNIP1 or **(C) **co-transfected with the indicated miRNAs and plasmid cDNAs. TNIP1 cDNA construct contained the complete coding sequence with a truncated 3'UTR. **(D) **Genome browser view of miR-517a/c binding site (red box) in TNIP1 3'UTR showing relative conservation across species. Also indicated is the presence of SNP rs72558377 in the binding site (red arrow). **(E) **Predicted base pairing between miR-517a and the TNIP1 binding site containing the major (C) and minor (T) alleles of rs72558377. '|' and ':' indicate Watson-Crick and wobble base pairing, respectively.(D) Both SNP alleles of the miR-517a/c target site in TNIP1 were cloned into the 3'UTR of a firefly luciferase reporter. The reporter also expressed *Renilla *luciferase from a separate promoter. The reporter was co-transfected with the indicated miRNAs in HEK293 cells. Firefly luciferase activity was normalized to that of *Renilla *luciferase. Statistical comparisons are relative to negative control (miR-neg). All values are means ± SD. B and C, n = 3. E, n = 4. *P ≤0.05, **P 0.01. miRNAs, micro RNAs; siRNAs, small interfering RNAs; SD, standard deviation; TNIP1, tumor necrosis factor alpha-induced protein 3 interacting protein 1.

miRNAs function primarily by down-regulating the expression of their targets. Therefore, we deduced the likely target of miR-517a/c was an inhibitor of NF-κB signaling. We searched the TargetScan predictions for known repressors. TNIP1, also known as A20-binding inhibitor of NF-κB activation (ABIN1), was one of the top predicted targets. TNIP1 binds to TNFAIP3, a ubiquitin-editing enzyme, and together they inhibit RIPK dependent NF-κB signaling [25,26]. A clone for the mouse Tnip1 gene was present in the cDNA library used for the analysis in Figure 2; however, it did not pass the multiple stages of filtering to be included in the final list of NF-κB modulators [20]. To test if TNIP1 was indeed a functional target of miR-517a/c, we measured its protein levels in HEK293 cells transfected with the miRNA mimics. Both miR-517a and miR-517c, but not miR-517b, potently reduced TNIP1 protein levels (Figure 5A). Next, we tested if siRNAs targeting TNIP1 could phenocopy miR-517a/c. Indeed, two independent siRNAs significantly increased endogenous expression of IL8 and TNF in HEK293 cells (Figure 5B). Finally, we hypothesized that overexpression of TNIP1 would block the effect of miR-517a/c. We tested this by creating a TNIP1 cDNA construct with a truncated 3'UTR lacking the miR-517a/c target site. This ensured that the effects we saw were due to the activity of TNIP1 protein and not due to competition for miR-517a/c binding. Expression of TNIP1 from the construct was confirmed via western blot (see Additional file 2, Figure S3). Co-transfection of the TNIP1 construct with the miRNA mimics reduced miR-517a/c-induced expression of IL8 and TNF in HEK293 cells (Figure 5C).

Next, we took a closer look at the genomic region corresponding to the miR-517a/c binding site in TNIP1. This revealed two interesting features. First, the target site is poorly conserved, with notable differences in the mouse and rat genomes (Figure 5D). Secondly, a SNP (rs72558377) is located in the target site corresponding to the third position of the miRNA seed (Figure 5E). The minor allele (T) results in a G-U wobble base pair, possibly affecting miRNA-target interaction (Figure 5E). To test the functional consequence of this SNP as well as demonstrate direct interaction between miR-517a/c and TNIP1, we cloned sequences containing the major and minor alleles immediately downstream of a firefly luciferase reporter. The same plasmid also expressed *Renilla *luciferase from a separate constitutive promoter, which was used as a control for transfection efficiency. miR-517a and miR-517c repressed the reporter containing the major allele variant but had no effect on the empty vector (Figure 5F). miR-517b also inhibited reporter activity, but to a lesser extent (Figure 5F). The repression by the miR-517 miRNAs was significantly alleviated in the minor allele reporter (Figure 5F). These results suggested that miR-517a/c interact directly with TNIP1 and that the minor allele of SNP rs72558377 decreases miRNA-target interaction and consequently miR-517a/c mediated repression of TNIP1.

Lastly, given that TNIP1 is a TNF regulated gene [27] and miRNAs are common in feedback regulation, we hypothesized that NF-κB regulates miR-517a/c which in turn activates the pathway through knockdown of TNIP1. To test this we treated HEK293 cells with TNF over time and measured expression of TNIP1 mRNA and miR-517a/c. While there was a significant increase in TNIP1 expression, miR-517a/c expression was not significantly changed (see Additional file 2, Figure S4).

### miR-517a/c induce apoptosis in HEK293 and U-2 OS cells

**Figure 6:**
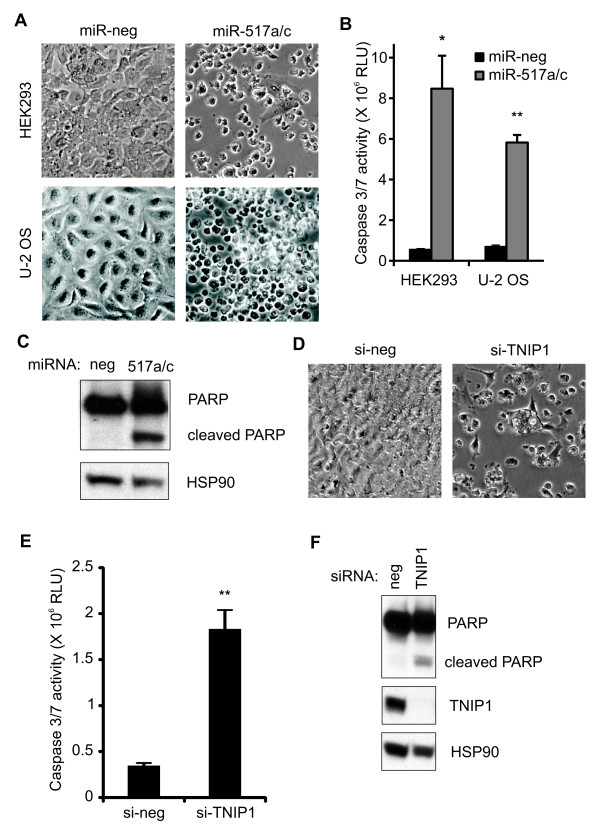
**miR-517a/c induce apoptosis in HEK293 and U-2 OS cells**. **(A) **Microscope images of HEK293 and U-2 OS cells transfected with miR-517a/c. **(B) **Caspase3/7 activity was measured in HEK293 and U-2 OS cells transfected with miR-517a/c with the Caspase-Glo 3/7 luminescence assay. **(C) **Western blot analysis of whole cell protein extracts from HEK293 cells transfected with miR-517a/c. Cleavage of PARP is evident by the lower molecular weight fragment and is indicative of caspase3 activity. HSP90 was the loading control. **(D) **Microscope images of HEK293 cells transfected with TNIP1 siRNA. **(E) **Similar to (B) casapase 3/7 activity in HEK293 cells transfected with pooled siRNAs against TNIP1. **(F) **Western blot analysis of whole cell protein extracts transfected with pooled TNIP1 siRNAs. All values are means ± SD. n = 3. *P ≤0.05, **P ≤0.01. miRNAs, micro RNAs; PARP, poly(ADP-ribose) polymerase; siRNAs, small interfering RNAs; SD, standard deviation; TNIP1, tumor necrosis factor alpha-induced protein 3 interacting protein 1.

miR-517a/c activation has been shown to promote cell survival and induce oncogenesis [28,29]. Surprisingly, we observed extensive cell death in an immortalized cell line, HEK293, and an osteosarcoma cell line, U-2 OS, transfected with miR-517a/c (Figure 6A). We measured caspase-3 (CASP3) and caspase-7 (CASP7) activity in cells transfected with miR-517a/c using the Caspase-Glo 3/7 luminescence assay. miR-517a/c increased CASP-3/7 activity 16-fold in HEK-293 cells and 8-fold in U-2 OS cells relative to the negative control (Figure 6B). To confirm the assay results, we tested the effect of miR-517a/c on the cleavage of poly (ADP-ribose) polymerase 1 (PARP1), an endogenous CASP3 target. miR-517a/c induced cleavage of full length PARP1 while no cleaved PARP1 was observed in lysates of control cells (Figure 6C). Together, these results suggested that miR-517a/c induced apoptosis in HEK293 and U-2 OS cells.

Next, we tested if miR-517a/c induced apoptosis was a result of TNIP1 knockdown. TNIP1 promotes cell survival and is essential for development. Mice lacking TNIP1 have extensive TNF induced apoptosis in the liver and few are born live [30]. Similar to miR-517a/c, siRNA mediated knockdown of TNIP1 increased cell death in HEK 293 cells (Figure 6D). Also, TNIP1 knockdown increased CASP-3/7 activity (Figure 6E) and cleavage of PARP1 (Figure 6F). Together, these results suggested that knockdown of TNIP1 contributes to the apoptosis inducing activity of miR-517a/c.

Finally, we questioned whether the apoptosis inducing effects of miR-517a/c were specific to cancer-derived/immortalized cell lines. We transfected primary HUVECs with miR-517a/c and, similar to HEK293 and U2-OS cells, we observed increased cell death and CASP-3/7 activity (see Additional file 2, Figure S5). Hence, these results suggest that miR-517a/c can induce apoptosis in primary and immortalized cells.

## Discussion

In this study, we identified 22 miRNAs that regulated basal or TNF-induced NF-κB signaling using high-throughput cell-based screening. Some of these hits are known regulators or direct targets of NF-κB. miR-24, miR-125b and miR-210 are direct NF-κB targets and were hits in our screen [31-33]. Further, miR-125b was recently shown to promote NF-κB signaling by targeting TNFAIP3 [16]. Likewise, miR-210 was recently shown to inhibit signaling by targeting NF-ΚB1/p50 [34]. Both of these findings are consistent with our screening data and suggest miR-125b and miR-210 are feedback regulators of NF-κB signaling. Also, studies by Jiang *et al. *suggest miR-30e-3p promotes NF-κB signaling by targeting the inhibitor IκB-α [35]. While miR-30e-3p did not meet the cutoff in our +TNF screen (*P *= 0.05), the related miR-30a-3p was a hit (*P *= 0.02), activating reporter activity. In all, these results confirmed that our primary screen effectively identified known miRNA regulators of NF-κB signaling.

During the preparation of this study, to our knowledge two other groups published screens investigating the role of miRNAs in NF-κB signaling [36,37]. A comparison of the experimental methods is detailed in Additional file 1, Table S4. The first by Lu *et al. *looked at the effects of miRNAs on basal NF-κB reporter activity (that is, non-stimulated) [36]. They found miR-301a was the strongest inducer (approximately 5-fold increase in reporter activity) and that it functioned by targeting NFKB repressing factor (NKRF). miR-301a was not a hit in our screen (*P *= 0.18; average fold change = 2.3). However, the related family members miR-130a and miR-130b (that is, with the same seed regions as miR-301a and likely overlapping targets) both increased basal reporter activity by 4.5-fold (*P *= 0.04) and 3.6-fold (*P *= 0.06), respectively (see Additional file 1, Table S2). Further, miR-130a was a hit in the +TNF screen with a 3.8-fold increase (*P *= 0.02; Table 1). Hence, our results are in agreement with the Lu *et al. *study suggesting that the miR-301/miR-130 family positively regulates NF-κB signaling. However, none of the activators confirmed in our secondary screen (miR-483, miR-517a and miR-517c) were hits in the Lu *et al. *study.

In the second study by Keklikoglou *et al.*, the authors examined the effect of miRNAs on TNF-induced NF-κB reporter activity [37]. They identified 13 miRNA families as hits (*P *< 0.2). Upon initial evaluation, none of these were hits in our screen. We then relaxed our *P*-value cutoff from 0.02 to 0.05 and three miRNA families overlapped between the screens: miR-27ab, let-7/miR-98 and miR-500. Interestingly, members of the miR-181 family consistently upregulated the reporter in the Keklikoglou *et al. *study, while all four members downregulated reporter activity in our study to varying degrees (see Additional file 1, Table S2). Our results are consistent with a recent study by Sun *et al. *demonstrating miR-181b inhibition of NF-κB through importin-α3 [38]. Further, miR-517a/c were identified as negative regulators in the Keklikoglou screen. miR-517a/c were potent activators in our -TNF screen and were not hits in our +TNF screen (Table 1). However, as noted before, we did observe a slight decrease in reporter activity by miR-517c in the secondary screen (Figure 1C). Interestingly, none of the validated hits from our +TNF secondary screen (miR-210, miR-375 or miR-483) were hits in the Keklikoglou *et al. *study.

We believe the differences in the screen results largely stem from methodological and technical differences (see Additional file 1, Table S4). All three studies used different NF-κB reporters that were transiently or stably expressed in HEK293/293FT cells. A critical factor may be the sensitivity and specificity of these reporters. Also, the duration of transfection was 72 hours in our screen, but 48 hours in the Lu and Keklikoglou screens. During the optimization of our screen, we found that this longer transfection period was necessary for the optimal impact of our positive controls on reporter activity (data not shown). However, the optimal transfection time depends on several factors including the abundance and half-life of the target proteins as well as the response kinetics of the reporter assay. In all, however, the differences between the screens are not unusual. Such discordance is also found in large RNAi screens [39,40]. This highlights the importance of replication studies and suggests that follow-up of top hits from all screens is necessary to understand fully the roles of diverse miRNAs in NF-κB signaling.

Determining the mechanism of miRNA action by finding their functional targets is challenging. This problem is two-fold. First, identify the targets, which can number in the hundreds, and second, from these, identify the ones mediating the effect of the miRNA. This can be limited by a lack of preexisting knowledge of the pathway of interest. Also, exhaustively testing the candidates can be unfeasible (miRNA hits times dozens to hundreds of candidates). We addressed this problem by integrating computational predictions from TargetScan and data from our genome-wide cDNA screen for regulators of NF-κB signaling [20]. With this method, we derived high confidence candidate targets for all the miRNAs with the exception of miR-517a/c. This was likely because miR-517a/c had about eight times fewer predicted targets than the other miRNAs (see Additional file 1, Table S3). For future studies, the target list from our analysis has to be validated experimentally. A few high-throughput experimental methodologies for evaluating miRNA targets have been successfully implemented. These include high-throughput sequencing of RNAs isolated by crosslinking immunoprecipitation (HITS-CLIP), photoactivatable-ribonucleoside enhanced crosslinking and immunoprecipitation (PAR-CLIP), ribosome profiling, and microarray analysis of cells or tissues with perturbed miRNA states [41-44]. These datasets are becoming available for more miRNAs and their integration into functional annotation pipelines, such as the one in our study, will be highly informative in identifying functional miRNA-mRNA interactions.

For follow up studies, we focused on the role of primate specific miR-517a and miR-517c as potent inducers of NF-κB signaling. We identified TNIP1, an inhibitor of NF-κB, as a direct target gene. Interestingly, miR-517b was not a hit in our screen despite extensive homology to miR-517a/c (Figure 4A). Also, miR-517b did not phenocopy miR-517a/c in secondary screens (Figure 3A) or knockdown TNIP1 protein expression (Figure 5A). These differences were likely due to a single nucleotide shift in the miR-517b mature sequence relative to miR-517a/c (Figure 4A). According to TargetScan predictions [45], this shift results in base-pairing between TNIP1 and nucleotides 2 to 7 of miR-517b as opposed to nucleotides 2 to 8 in miR-517a/c-the latter resulting in a more favorable miRNA-target interaction. This result highlights the importance of the miRNA seed in mediating miRNA-target interactions and suggests caution in extrapolating the function of individual miRNAs to their family members, particularly when their seed regions differ. Interestingly, during the preparation of this study we examined sequence reads of the miR-517b locus from miRBase [46], the central repository for miRNA sequences, and found a variant identical to miR-517a. This suggests that the shifted variant is either a result of read errors or a true 5' isomiR-the likes of which have been demonstrated in multiple species [47]. We informed the curators of miRBase, and the unshifted sequence (identical to miR-517a) is the new reference for miR-517b (now miR-517b-3p) as of miRBase v17.

While knockdown of TNIP1 was sufficient to induce expression of TNF and IL8 as much as 18- and 56-fold, respectively (Figure 5B), miR-517a/c were more potent inducers (Figure 4B). Also, overexpression of TNIP1 did not completely block miR-517a/c induced expression of TNF and IL8. This suggests that the effect of miR-517a/c on NF-κB signaling may be mediated by other genes in the pathway in addition to TNIP1. This is consistent with numerous studies suggesting that miRNAs regulate cellular pathways by targeting multiple components [48]. Further studies are needed to elucidate additional miR-517a/c targets. The TNIP1 3'UTR harbors a functional SNP in the miR-517a/c binding site. SNPs are the most common form of human polymorphisms with millions validated in the National Center for Biotechnology Information (NCBI)'s dbSNP. Here, we show that the minor allele of SNP rs72558377 (T) creates a G-U base pair between miR-517a/c and TNIP1 that disrupts binding, resulting in reduced knockdown of TNIP1. This is consistent with studies demonstrating the deleterious effect of G-U base pairing between a miRNA's seed and target mRNA [49,50]. There are currently no reported phenotypes associated with rs72558377, but future studies may shed light on the potential consequence of this SNP and the broader biological function of miR-517a/c.

An oncogenic role for miR-517a has been suggested in liver and brain tumors [28,29]. Given miR-517a/c activated NF-κB signaling in our screen, this is consistent with the well-established anti-apoptotic role of NF-κB [51,52]. However, we found miR-517a/c induced apoptosis in the immortalized cell line HEK293, in the osteosarcoma cell line U-2 OS and in primary HUVECs. Corroborating our results, a study by Yoshitomi *et al. *found miR-517a induced apoptosis in the bladder cancer cell lines T24 and BOY [53]. Also, interestingly, Na *et al. *recently found miR-517a down-regulated in the placenta of patients with complete hydatidiform moles-a condition characterized by increased trophoblast proliferation [54]. Hence, our results suggest that the knockdown of TNIP1 by miR-517a/c may both activate NF-κB signaling and repress its anti-apoptotic activity. Also, these conflicting functions of miR-517a/c may reflect the complex role of NF-κB in the regulation of cell survival. In some cases, NF-κB can promote apoptosis in a context and cell type-specific manner [55]. Additional studies are needed to understand how survival signals differ in various cell types in response to miR-517a/c and the role NF-κB may play in this process. As miR-517a/c are suppressed in most cell/tissue types, their reported activation by demethylating drugs, such as 5-aza-2′-deoxycytidine (5-aza-dc) [56] may have therapeutic value in the treatment of certain cancers.

## Conclusions

This study provides a valuable resource for the functional annotation of miRNAs in the NF-κB pathway. Our data suggest that miRNAs are common regulators. While additional studies are needed to identify the functional targets of these miRNAs, we provide a starting list of likely candidates by integrating target predictions and cDNA screening data. Lastly, we conclude that miR-517a/c are pro-apoptotic and potent activators of NF-κB that function at least in part through TNIP1.

## Methods

### Tissue culture and cell maintenance

HEK293 andU-2 OS cells were purchased from American Type Culture Collection (ATCC, Manassas, VA, USA). Primary HUVEC cells (Lonza, Walkersville, MD, USA) were a kind gift from Dr. Vladimir Muzykantov (University of Pennsylvania) and were used no later than passage 6. The NF-κB-luc (HEK293) cell line was a generous gift from Dr. Sumit Chanda (Burnham Institute). Briefly, HEK293 cells were transfected with 2 μg of a 5X NF-κB-luc luciferase reporter in a 100 mm dish and clonal selection was carried out with neomycin. HEK293 and U-2 OS cells were maintained in growth media containing 10% fetal bovine serum (FBS), 1X penicillin/streptomycin/glutamine (Life Technologies, Grand Island, NY, USA) in (D)MEM. HUVECs were maintained in growth media made with the EGM-2 BulletKit (Lonza; CC-3162) according to the manufacturer's protocol. All cells were kept in a humidified incubator at 37°C and 5% CO_2_.

### siRNAs and miRNA

All Ambion miRNA mimics used in the primary screen and follow-up studies as well as siRNAs against p65 (#216912 and #143124) were from Life Technologies. siRNAs against TNIP1 (SI04315815 and SI04317341) were from Qiagen (Valencia, CA, USA).

### Primary screen

A miRNA mimic library (Ambion; Pre-miR library v 8.0) was spotted at 1.6 pmol/well in a 384-well format. NF-κB-luc cells were reverse transfected with Lipofectamine2000 (Life Technologies). Briefly, 10 μL of OptiMEM (Life Technologies) was added to each spotted well. A total of 10 μL of transfection reagent mix containing 1% Lipofectamine2000 in OptiMEM was then added to each well and incubated for 20 minutes. Cells were prepared in 2X growth media ((D)MEM, 20% FBS, 2X L-glutamine (Life Technologies)) at a final concentration of 1.5 × 10^5 ^cells/mL. A total of 20 μL of cell suspension was added to each well. Plates were maintained under normal growth conditions for 72 hours. Stock TNF (Sigma, St. Louis, MO, USA) solution was diluted in (D)MEM and 5 uL was added to each well to a final concentration of 40 ng/mL. Luciferase levels were quantified 8.5 hours later, using BriteLite assay systems (Perkin Elmer, Shelton, CT, USA) on an Envision Multilabel plate reader (Perkin Elmer) following the manufacturer's protocols. Raw luminescence values were log transformed and robust *Z*-scores were calculated for each well using the following formula: *Z*-score = (x_i _-med(x))/mad(x). Where x_i _is the luminescence value for well i, med(x) is the median of the plate and mad(x) is the median absolute deviation of the plate. *P*-values were estimated with a Wilcoxon rank sum test (two-sided) comparing each duplicate pair to the experiment wide distribution.

### Secondary screen

Secondary screen was performed as per the primary screen in a 96-well format (final volume 100 μL). NF-κB-luc cells were co-transfected with the miRNA mimics (50 nM final) and pGL4.74 *Renilla *construct (Promega, San Luis Obispo, CA, USA; final 30 ng/well). Following treatment with TNF (40 ng/mL-final), firefly and *Renilla *luciferase levels were quantified using the Dual-Glo Luciferase Assay System (Promega) according to the manufacturer's protocol.

### Caspase-Glo assay

HEK293 and HUVEC cells were plated in a 96-well plate at 5,000 cells/well in 100 μL of the respective growth media with no antibiotics. The next day, cells were transfected with miRNA mimics at a final concentration of 50 nM with Lipofectamine RNAiMAX (Life Technologies) according to the manufacturer's protocol. Caspase activity was measured 96 hours later with the Caspase-Glo 3/7 Assay (Promega) according to the manufacturer's protocol.

### Computational analysis for high-confidence miRNA targets

miRNA targets were predicted using Perl scripts from TargetScanHuman (http://www.Targetscan.org) version 6.0 as per the websites instructions [21,22]. Predictions were made irrespective of target site or miRNA conservation. The 328 miRNA sequences used as input for the program are listed in Additional file 1, Table 1 and correspond to the miRBase v 8.0 annotation used in the construction of the library. For the cross-screen comparisons, the list of modulators was derived from a previously described study [20]. All subsequent analyses were done in R.

### Transfection and TNF stimulation of HEK293, U-2 OS and HUVEC cells

HEK293 and U-2 OS cells were seeded in 6-well (for protein) or 24-well (for RNA) plates at 1 × 10^5 ^and 2.5 × 10^4 ^cells/well, respectively, in media containing 10% FBS and 1X L-glutamine in OptiMEM/(D)MEM (1:1). HUVEC cells were seeded in 60 mm dishes (for protein) or 24-well plates (for RNA) at 1.6 × 10^5 ^and 4 × 10^4 ^cells/well respectively, in HUVEC growth medium without antibiotics. Cells were co-transfected the next day with plasmids and miRNAs using Lipofectamine 2000 (Life Technologies) according to the manufacturer's protocol. RNAiMAX was used for all other transfections according to the manufacturer's protocol. miRNAs and siRNAs were transfected at a final concentration of 50 nM each. Plasmids were transfected at a final concentration of 125 ng/well (24-well plate) or 500 ng/well (6-well plate). Mimic 'miR-517a/c' contained equal amounts of miR-517a and miR-517c. The amount of nucleic acid was held constant across conditions in every experiment by adding the respective negative controls (negative control siRNAs/mimics or empty vector). Cells were transfected for 72 hours (unless otherwise stated in the text) and maintained under normal growth conditions before harvesting for protein or RNA. HEK293 cells were stimulated with TNF at a final concentration of 40 ng/mL for 3 hours (unless otherwise indicated) before processing for RNA.

### Western Blot

Whole cell protein extracts were isolated from HEK293 cells with ice-cold RIPA buffer. Nuclear and cytoplasmic extracts were isolated with the NE-PER Nuclear and Cytoplasmic Extraction kit (Pierce, Rockford, IL, USA). All extraction buffers were supplemented with a protease inhibitor cocktail (Complete; Roche, Indianapolis, IN, USA). Protein concentrations were quantified with the DC protein assay (BioRad, Hercules, CA, USA). A total of 50μg (whole cell) or 10μg (nuclear/cytoplasmic extracts) of protein was resolved on Tris-HCL SDS gels (BioRad) and transferred to polyvinylidene difluoride (PVDF) membranes. Membranes were blocked for 1 hour at room temperature in blocking solution (2.5% milk, 0.5% Tween20, 1X Tris-buffered saline) followed by a 1 hour incubation with the primary antibody in blocking solution. Primary antibodies used were: anti-p65 (4764S), anti-IκB-α (4814S), anti-Lamin A/C (2032), anti-PARP (9532S), anti-HSP-90 (4875; Cell Signaling Technologies, Danvers, MA, USA), anti-Tubulin (ab7291; Abcam, Cambridge, MA, USA), anti-TNIP1 (15104-1-AP; Proteintech Group, Chicago, IL, USA) and anti-GAPDH (sc-25778; Santa Cruz Biotechnology, Santa Cruz, CA, USA). Membranes were rinsed twice each with TBS-0.1% tween and blocking solution then probed with anti-rabbit immunoglobulin G (IgG) horseradish peroxidase (HRP)-linked secondary antibody (NA934-1ML; GE Healthcare, Mickleton, NJ, USA) for 30 minutes in blocking solution. Membranes were rinsed 4 times for 15 minutes in TBS-0.1% tween solution followed by the application of enhanced chemiluminescence (ECL) western blotting detection reagent (GE Healthcare) and developed by standard autoradiograph techniques.

### p65 ELISA assay

p65 activation was measured in nuclear extracts of HEK293 cells with a commercial DNA binding ELISA kit (ActiveMotif (Carlsbad, CA, USA) TransAM NFκB p65 Chemi) according to the manufacturer's protocol. A total of 500 ng of protein was used for each assay.

### Cloning of TNIP1 construct

Human full-length TNIP1 cDNA (BC012133; Thermo Scientific, Waltham, MA, USA) was PCR amplified with primer set 5'-GCATACTAGTAAGCTCCCCGTCCTCGGCCA-3' and 5'- GCATCTCGAGAGCCTCTCATCCAGCTGAGGCTCT-3' (Integrated DNA Technologies, Coralville, IA, USA) and cloned into the SpeI and XhoI restriction sites of pCMV-sport6 vector (Invitrogen) using standard recombinant DNA techniques. The resulting construct was verified by DNA sequencing. Expression of TNIP1 from the construct was confirmed by western blot.

### Cloning of 3'UTR reporter constructs

Both strands of the respective target regions were synthesized (Intergrated DNA Technologies) with overhangs for ligation into the NheI and XhoI restriction sites of the pmiRGlo Dual Luciferase vector (Promega). Complementary strands were annealed by mixing 50 μL of each 100 μM oligo (in 1x Tris-EDTA (TE)) with 100 μL of 2X annealing buffer (100 mM NaCl in 1X TE) and placing in a 99°C water bath for 2 minutes. The heat source was then turned off and the samples were allowed to cool to room temperature. Annealed oligos were then purified with the Qiaquick Nucleotide removal kit (Qiagen). The resulting double stranded approximately 46 bp DNA segment was diluted (1:100) and cloned into the restriction sites of the pmiRGlo vector using standard recombinant DNA techniques. The resulting constructs were verified by DNA sequencing. The oligos synthesized were as follows: TNIP1 major allele sense and antisense (5'-CTAGATCCCTGGATAGCTATTTGCACGAATCATGGACATAAATCCA-3', 5'- TCGATGGATTTATGTCCATGATTCGTGCAAATAGCTATCCAGGGAT-3'), TNIP1 minor allele sense and antisense (5'-CTAGATCCCTGGATAGCTATTTGCATGAATCATGGACATAAATCCA-3', 5'- TCGATGGATTTATGTCCATGATTCATGCAAATAGCTATCCAGGGAT-3').

### RNA extraction and quantitative PCR

RNA was extracted from HEK293 cells in high-throughput format with the Nucleospin 96 RNA kit (Macherey-Nagel, Bethlehem, PA, USA) following the manufacturer's protocol. For the TNF time-course experiment, RNA was extracted with miRNeasy kit (Qiagen) following the manufacturer's suggested protocol for total RNA isolation (including small RNAs). cDNA was generated from 500 ng of RNA using the miScript Reverse Transcription kit (Qiagen) and quantitative PCR was performed on the 7900HT Real-Time PCR System (Life Technologies) using the Quantitect SYBR Green PCR kit (Qiagen) according to the manufacturers' protocols. The ΔΔCT method was used for relative expression quantification using the RQ manager software v2.4 (Life Technologies). 18S rRNA (or RNU6B) was the endogenous reference for all mRNA (or miRNA) quantification experiments. All primer sets were purchased from Qiagen: TNF (QT01079561), IL8 (QT00000322), IL6 (QT00083720), TNIP1(QT00044072), 18S rRNA(QT00199367), miR-517a (MS00004459), miR-517c (MS00009954), RNU6B (MS00029204).

### Statistical analysis

All comparisons between group means were performed with a two-sided heteroscedastic Student's t-test.

## Abbreviations

bp: base pair; CASP3: caspase 3; CASP7: caspase 7; CS: context score; (D)MEM: (Dulbecco's) modified Eagle's medium; EDTA: ethylenediaminetetraacetic acid; ELISA: enzyme-linked immunosorbent assay; FBS: fetal bovine serum; HRP: horseradish peroxidase; HUVECs: human umbilical vein endothelial cells; IκB: I kappa-B; IgG: immunoglobulin G; IKK: I kappa-B kinase; IL: interleukin; LPS: lipopolysaccharide; mad: median absolute deviations; miRNAs: micro RNAs; ncRNAs: non-coding RNAs; NF-κB: nuclear factor kappa-B; NF-κB-luc: nuclear factor kappa-B responsive luciferase; NKRF: nuclear factor kappa-B repressing factor; PARP1: poly(ADP-ribose) polymerase 1; PCR: polymerase chain reaction; siRNAs: small interfering RNAs; SNP: single nucleotide polymorphism; TE: Tris-ethylenediaminetetraacetic acid; TNF: tumor necrosis factor; TNIP1: tumor necrosis factor alpha-induced protein 3 interacting protein; UTR: untranslated region.

## Competing interests

The authors declare that they have no competing interests.

## Authors' contributions

AO and YW performed and designed screen optimization and screen. All other experiments were performed by AO. MAE and LA aided in the design of the study. JBH conceived of the study and guided the design of experiments. AO and JBH drafted the manuscript. All authors read and approved the final manuscript.

## Supplementary Material

Additional file 1 **Supplemental tables**. Table S1. miRNA library annotation; Table S2. Primary screen data; Table S3. Number of predicted targets per miRNA hit; Table S4. Comparison of genome-wide miRNA screens for regulators of NF-κB. Click here for file

Additional file 2 **Supplemental figures**. Figure S1. p65 siRNA validation; Figure S2. Functional analysis of miR-517a/c in primary HUVECs; Figure S3. TNIP1 overexpression in HEK293 cells; Figure S4. TNF time course treatment in HEK293 cells; Figure S5. miR-517a/c-induced apoptosis in HUVECs. Click here for file
