# Supplementary material for: A functional genomics screen for microRNA regulators of NF-kappaB signaling
Source: BMC Biol. 2013 Feb 28;11:19. doi: 10.1186/1741-7007-11-19 (PMC3621838; doi:10.1186/1741-7007-11-19)
Supplement: Additional file 2 — Supplemental figures. Figure S1. p65 siRNA validation; Figure S2. Functional analysis of miR-517a/c in primary HUVECs; Figure S3. TNIP1 overexpression in HEK293 cells; Figure S4. TNF time course treatment in HEK293 cells; Figure S5. miR-517a/c-induced apoptosis in HUVECs. [file 1741-7007-11-19-S2.PDF]

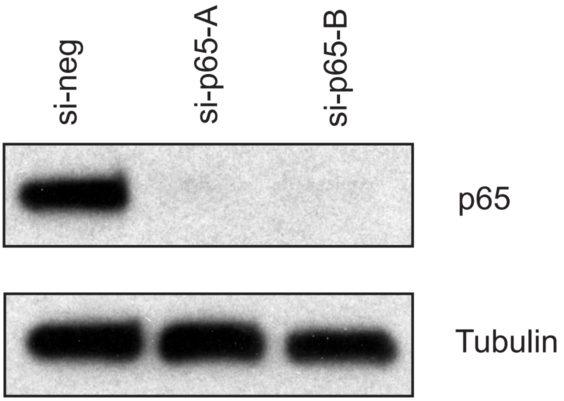

Figure S1. siRNA knockdown of p65 in HEK293 cells.

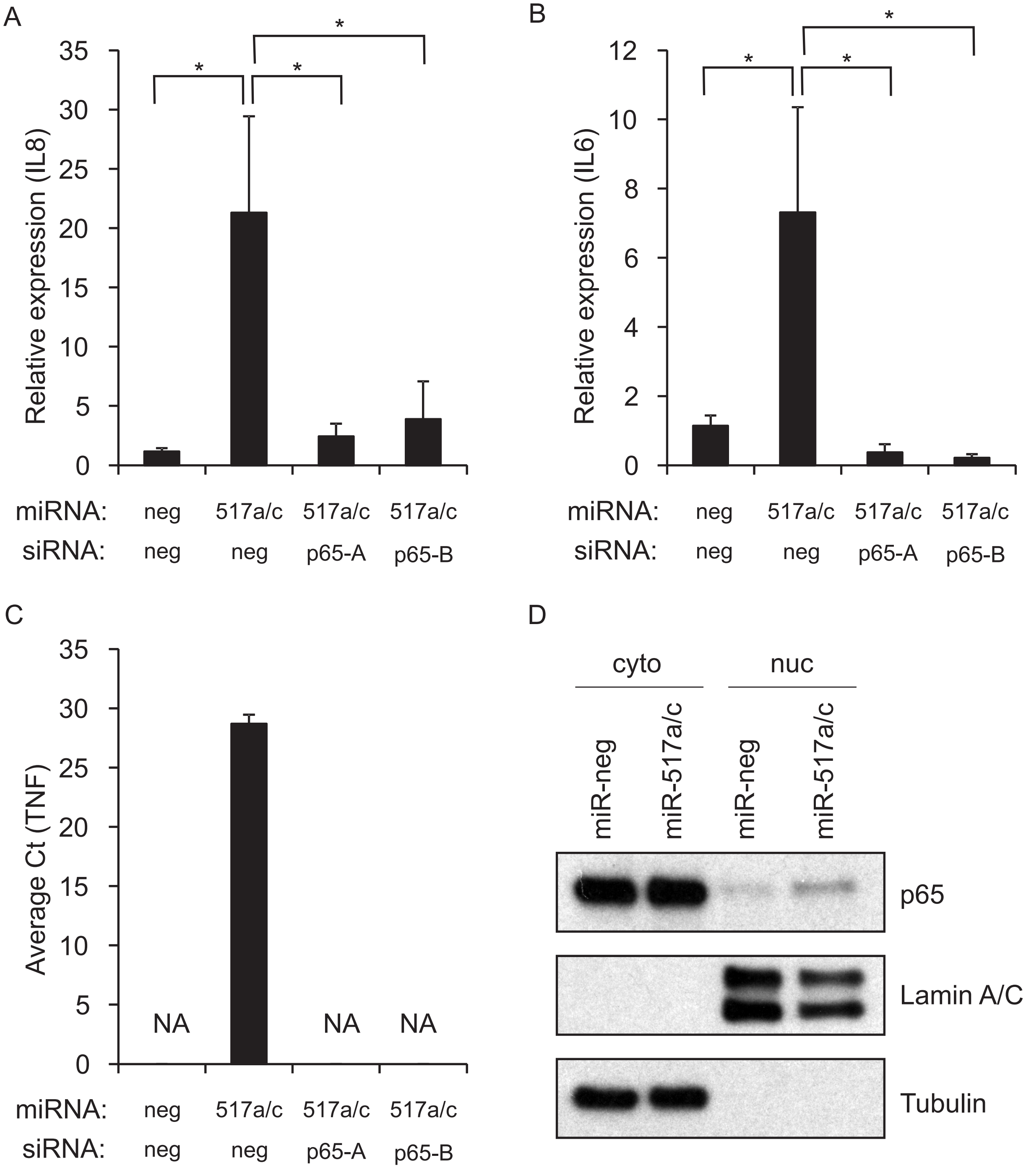

Figure S2. Functional analysis of miR-517a/c in primary HUVECs. (A-C) Expression of NF-KB targets IL8, IL6 and TNF in HUVECs transfected with the indicated miRNAs and siRNAs. (D) Western blot of cytoplasmic and nuclear extracts from miR-517a/c transfected HUVECS showing increased p65 in the nucleus. Lamin A/C and Tubulin are nuclear and cytoplasmic loading controls, respectively. n =4 for A-C. * p <= 0.05. NA = no amplification.

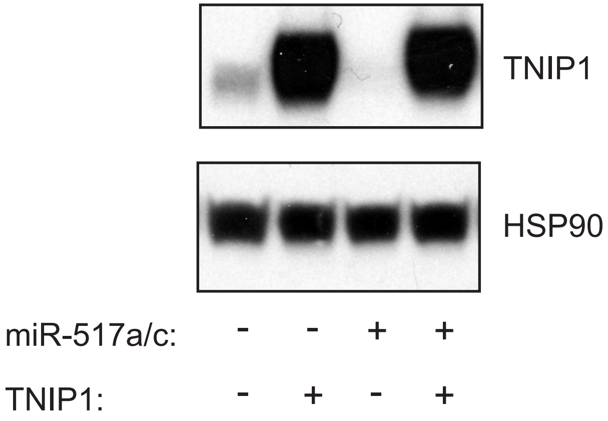

Figure S3. TNIP1 overexpression in HEK293 cells. TNIP1 construct lacking the miR-517a/c binding site was transfected into HEK293 cells along with miR-517a/c as indicated.

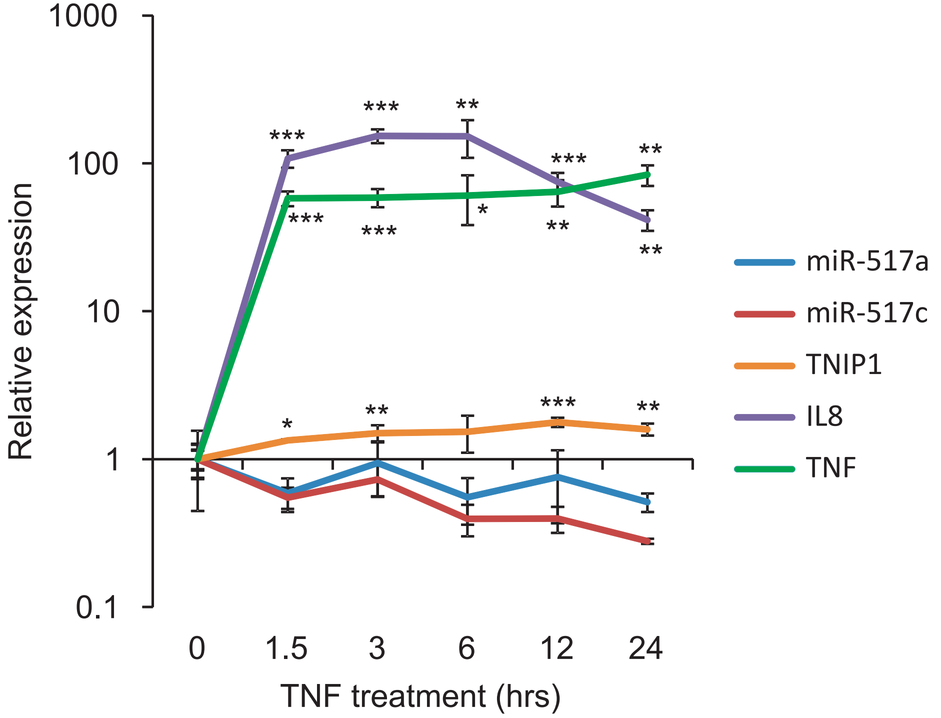

Figure S4. TNF time course treatment in HEK293 cells. HEK293 cells were treated with 40ng/mL of TNF for the indicated times and the expression of miR-517a/c, TNIP1 IL8 and TNF were measured via qPCR. y-axis is in log scale. n=4, *p<=0.05, ** p<=0.01, ***p <= 0.001

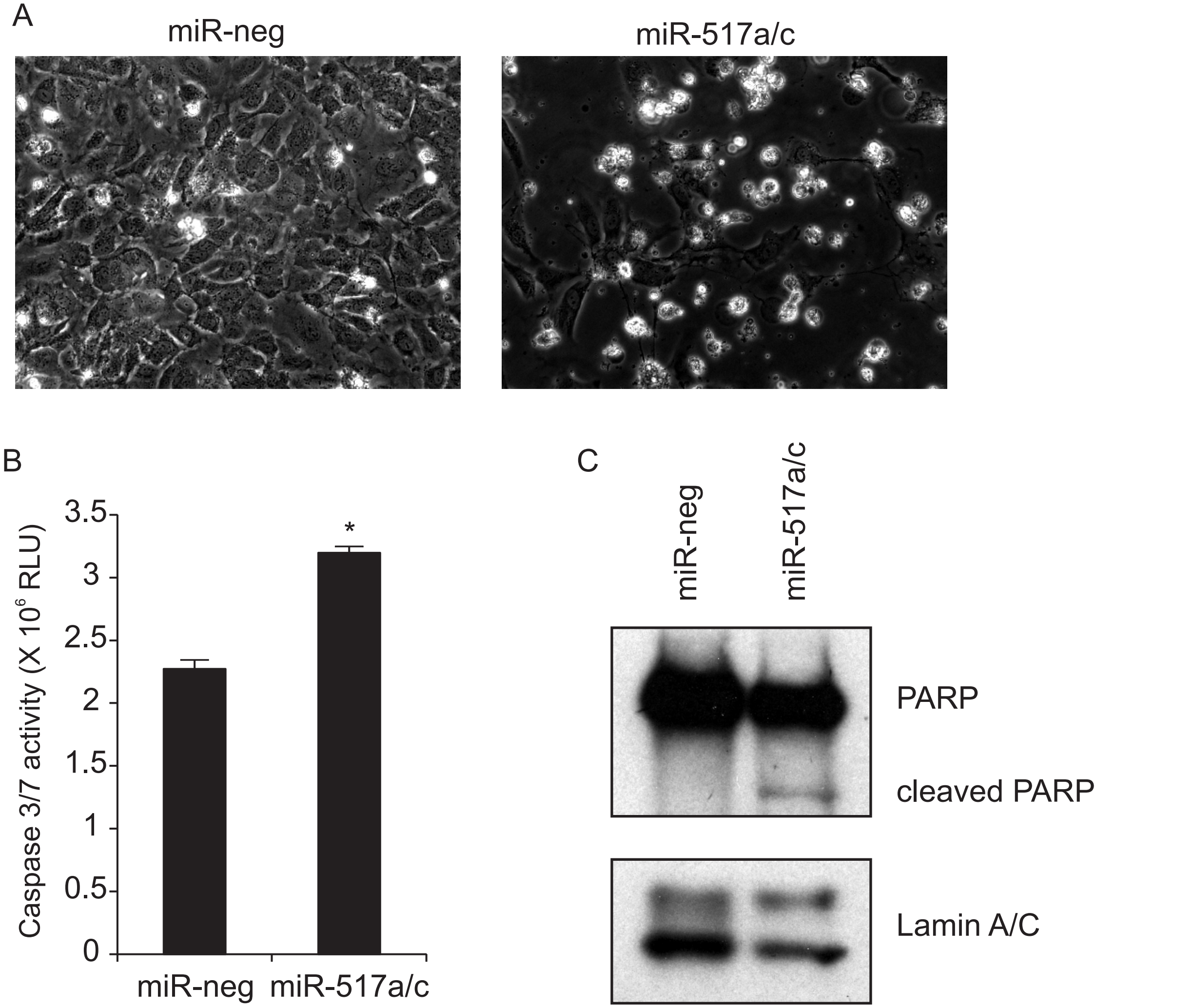

Figure S5. miR-517a/c-induced apoptosis in HUVECs .(A) Microscope images of HUVECs transfected with miR-517a/c. (B) Caspase3/7 activity was measured in HUVECs transfected with miR-517a/c with the Caspase-Glo 3/7 luminescence assay. (C) Western blot analysis of nuclear extracts from HUVECs transfected with miR-517a/c. Cleavage of PARP is evident by the lower molecular weight fragment and is indicative of caspase3 activity. Lamin A/C was the loading control. n=3 (B), *p<= 0.05
